# Interpreting and Reframing the Appropriate Adult Safeguard

**DOI:** 10.1093/ojls/gqab029

**Published:** 2021-09-24

**Authors:** Roxanna Dehaghani

**Keywords:** appropriate adults, human rights, vulnerable suspects, Police and Criminal Evidence Act 1984

## Abstract

The appropriate adult (AA) safeguard is an important procedural safeguard that can be implemented to protect vulnerable suspects at the police investigative stage of the criminal process. The safeguard is available for young suspects (below the age of 18) and adult suspects who are defined as vulnerable, and can be performed by a vast array of individuals. It is intended to protect evidence, enable effective participation and avoid miscarriages of justice. However, the safeguard lacks an underpinning conceptual framework; it is, and can, be interpreted in multiple ways, thus undermining its efficacy. Drawing upon doctrinal and socio-legal analysis, this article examines how the safeguard is—and, crucially, can be—conceptualised. It is argued that, although it is used principally as an evidential safeguard, the appropriate adult could be reimagined through the United Nations Conventions on the Rights of Persons with Disabilities and the United Nations Conventions on the Rights of the Child, with a specific focus on allowing for effective participation of the vulnerable suspect.

## 1. Introduction

The Police and Criminal Evidence Act (PACE) 1984, implemented in 1986, provided a legislative framework through which to protect suspects’ rights and entitlements, and to regulate police powers and procedures. This legislative framework introduced through Codes of Practice, namely Code C,^1^ the requirement for vulnerable suspects (children and young people below the age of 18^2^ and adults with a mental health condition or mental disorder^3^ who meet the requirements of a ‘functional test’^4^) to be provided with an appropriate adult (AA, or the safeguard). The functional test is intended to assess the suspect’s understanding and communication (of processes, rights and entitlements, and/or the significance of comments, questions and replies), suggestibility, compliance, and/or tendency towards providing unreliable, misleading or incriminating information (without knowing that they are doing so or wishing to do so) and/or being confused or unclear about their position.^5^

An AA should provide support, advice and assistance in relation to any aspect of Code C or any other Code of Practice, or when the vulnerable suspect is ‘given or asked to provide information or participate in any procedure’.^6^ The AA must also observe the propriety and fairness of police actions in relation to the suspect’s rights and entitlements, assist the suspect in his or her communication with the police whilst respecting the suspect’s right to silence, and ensure that the suspect has understood his or her rights and entitlements whilst also ensuring ‘that those rights are protected and respected’.^7^ Further, the AA should also be present for procedures such as charge, cautions, warnings in relation to adverse inferences, the taking of samples (ie fingerprints, photographs and DNA), reviews of detention and the conduct of intimate searches.^8^

The AA’s role—along with the definitions of vulnerability—has, however, evolved over time: who can perform the role, and for whom, has changed in some ways quite significantly since 1986.^9^ Yet, the underpinning purpose of the AA has not been subject to much debate, despite there being significant problems with how the safeguard is used, for whom it is used and why it is used (or not, as is often the case). This article marks an advance on current understandings of the safeguard by offering distinct approach(es) to conceptualisation, which situate the suspect’s rights and entitlements at the forefront. Without such a conceptualisation, it is possible that the AA’s role is co-opted to secure evidence, often at the expense of the suspect. Further, understanding the purpose of the safeguard is imperative when assessing the effectiveness of the safeguard as one must first understand what the safeguard *aims* to achieve before assessing whether it *has* indeed achieved this.

A clear purpose may also engender commitment to the regime through, and by, key stakeholders, such as the police, the courts, AA practitioners, managers and commissioners, legal representatives, healthcare professionals and suspects. Without grappling with the underpinning purpose of the safeguard, it is difficult—if not impossible—to argue in favour of compliance; such actors must understand why the safeguard is there, what it aims to achieve and/or whether its purpose has been fulfilled. Without a clearly defined purpose, the safeguard may be interpreted in multiple and potentially conflicting ways. As the police station is essentially becoming the site of the trial as fewer and fewer cases reach the courts,^10^ it is imperative that procedural safeguards at the pre-trial stage provide the necessary protection to suspects.

In section 2, this article will examine how the AA safeguard is conceptualised by examining and critiquing the evidential origins, current AA framework and existing case law. These, it will be argued, do not provide an unambiguous conceptualisation of the safeguard, but rather point towards the AA as an evidential safeguard, ie one that protects the integrity of the evidence. Further, the AA safeguard, as is examined herein, was not, and still is not, designed to meet the specific needs of vulnerable suspects. In section 3, drawing upon existing research, the article will consider how the safeguard for vulnerable suspects has thus far been constructed. Yet, it will be argued that the research does not fully examine the implications of the models put forth. Thereafter, in section 4, it will be argued that the safeguard could be reconceptualised through equality and human rights provisions in order to adequately protect vulnerable suspects. In doing so, the possibilities and limitations of equality and human rights approaches will be identified.

## 2. Evidential Origins and Contemporary Context

In this section, the evidential origins of the safeguard will be explored, in addition to the current legislative framework. The contention, as will be set out below, is that the safeguard is principally one of evidential protection.

### A. Evidential Origins

PACE and its accompanying Codes of Practice (which, as noted above, include the requirement for the AA safeguard) emerged from noted deficiencies in the Judges’ Rules. The Rules were first introduced in 1912 and provided guidance to the police on the various procedures and practices to be followed when detaining and questioning suspects,^11^ detailing what the police were permitted (or not) to do to ensure the admissibility of evidence at court. Failure to follow the rules could render the evidence inadmissible. However, problems with the Judges’ Rules were evidenced after three vulnerable individuals—an 18-year-old adult who was deemed ‘mentally subnormal’ and two children (aged 14 and 15) one of whom had educational difficulties and the other of whom spoke English as a second language—were wrongfully convicted of various offences relating to the death of Maxwell Confait.^12^ An inquiry^13^ into the case and the Judges’ Rules, which focused on the nature of police questioning of children and the ‘educationally subnormal’, found the Rules to be inadequate when protecting vulnerable suspects.^14^ The Royal Commission of Criminal Procedure (RCCP) (1979–81)^15^ was subsequently formed, leading to the introduction of PACE.^16^ The resultant legislative framework—PACE and its accompanying Codes of Practice—was designed to achieve ‘fairness, openness and workability’.^17^ Importantly, PACE included enhanced safeguards for vulnerable suspects under the guise of the AA.

Yet, two problems emerge within this framework: PACE focuses its attention on safeguarding the evidence in a similar vein to the Judges’ Rules (it is no coincidence that it is called the Police and *Criminal Evidence* Act) and, in doing so, does not adequately protect the suspect. Within this framework, the tasks of identifying vulnerability and implementing the safeguard are left with the police and the remedy for breach involves exclusion of evidence at trial, which in itself requires that a trial will take place. Further, a ‘one safeguard fits all’ approach was taken in response to multiple vulnerabilities. These contentions will be explored in more detail through examination of the contemporary context below.

### B. PACE and Code C

The first problem is that the PACE framework does not contain any information on vulnerability or the AA safeguard.^18^ Instead, the provisions are contained within the Codes of Practice, which, as soft law, can present obstacles to compliance and enforceability.^19^ As the AA safeguard is contained within the Codes rather than in PACE itself, failure to implement the AA safeguard—or a safeguard that is ‘appropriate’^20^—cannot result in civil or criminal sanction.^21^ Rather, the only remedy offered by the PACE framework is exclusion of evidence at trial, through sections 76 and 78.^22^ The first rule relates to confession evidence alone: to be excluded, the confession must have been obtained through oppression or otherwise rendered unreliable through things said or done,^23^ which can include breaches of the Codes of Practice where such breaches render the confession unreliable. The second rule allows any evidence to be deemed inadmissible should it adversely impact upon the fairness of the proceedings, although such determinations are made at the judge’s discretion.^24^ Within this framework, therefore, the question rests not simply with breach of the Codes, but with whether this breach has amounted to unreliability or unfairness; the concern is not for the suspect, but for the integrity of the evidence.

The manner in which vulnerability has been defined also lends support to an evidential contention. Prior to 2018, Code C made no mention of the importance of the AA to securing the suspects’ rights and entitlements. Rather, the definition of vulnerability in respect of adult suspects was on the ability to ‘understand the significance of what is said, of questions or of their replies’.^25^ Further, the rationale for protecting vulnerable suspects was focused on their ability to provide reliable evidence—those who were vulnerable were thought to ‘without knowing or wishing to do so, be particularly prone in certain circumstances to provide information that may be unreliable, misleading or self-incriminating’.^26^ The focus was on the provision of reliable and accurate information, and there was little to no recognition of the broader process such as, *inter alia*, the understanding of, and ability to enforce, rights and entitlements. In July 2018, the definition of vulnerability was expanded in respect of adult suspects (as above). The ‘functional test’ introduced does not apply to children, who are recognised as vulnerable on the basis of age alone, but is now imperative in the assessment of vulnerability in adults.^27^ Whilst the test includes a greater range of ‘vulnerabilities’—to include an explicit recognition of rights and entitlements and an acknowledgment of suggestibility and compliance—than the previous Code, which focused on reliability of evidence alone, the determination still rests with the custody officer, who is nevertheless a police officer.^28^

As noted in the Introduction, Code C also provides detail on the role and purpose of the AA. The AA should: provide support, advice and assistance in relation to various procedures; ensure that the police are acting fairly and advise an officer of at least Inspector rank if they are not; facilitate communication whilst respecting the right to silence; and ensure that the suspect’s ‘rights are protected and respected’.^29^ It was not until the changes in 2018 that the AA’s role involved any appreciation of the suspect’s rights,^30^ and it was also not until these changes that the right to silence was explicitly acknowledged in Code C.^31^ Nevertheless, there remain significant obstacles that undermine the AA’s ability to protect the suspect, as will be explored below.

The AA is required to facilitate communication, yet although the AA does not destroy privilege, they are not subject to it either,^32^ and could therefore be compelled to give evidence against the suspect at trial. The absence of legal privilege may undermine the safeguard by discouraging open lines of communication between the suspect and the legal representative where the AA is present or, where not present, by preventing the AA from gaining knowledge and awareness of the discussions between the suspect and their legal representative, which, of course, may include an explanation of the advice offered by the legal representative. This may seriously hinder the AA’s involvement in a police interview setting. Further, AAs are not always present for each and every procedure, particularly for adult suspects, and the interview tends to be the principal focus.^33^ At interview, the AA is also discouraged from taking an active role in that he or she cannot be unreasonably obstructive during interview, otherwise the AA can be asked to leave.^34^ This may also sit in tension with the suspect’s right to silence: the AA could be construed as ‘obstructive’ if reminding the suspect of his or her right to silence or—as a recent case demonstrated^35^—the right to legal advice. The problem here is that the Code does not elaborate on what constitutes an unreasonable obstruction, and although this could be beneficial from a police perspective (as they will have met their Code C obligations by calling out an AA,^36^ even if that AA is nothing more than ‘wallpaper’^37^), by failing to provide clarity regarding when the AA is and is not permitted to intervene, it may not ensure that the suspect is adequately protected. The AA’s presence can thus protect the integrity of the evidence, but may be ineffectual when assisting the suspect. It could be argued that such issues are resolved at court, yet this relies upon the case reaching court and the court then determining that the evidence is inadmissible.

### C. Case Law, the Courts and the AA

The manner in which the AA safeguard is implemented and indeed understood is also partly influenced by the case law. As noted above, under the PACE framework, the courts are responsible for deciding whether evidence should be admitted; it is through the rules of admissibility that compliance with the AA safeguard is achieved.^38^ In the limited case law available, the courts have explored the definition of vulnerability and the purpose of the AA safeguard. In *Weekes*, for example, young suspects were said to require an AA because they may ‘sometimes say things they do not mean’.^39^ The AA is someone who is ‘more experienced’ and should be present to assist the young person.^40^ In *Weekes*, the appellant had ‘entered into the realm where fairness demanded that [he] was supported by somebody older who could assist him in answering the questions or advising him if he could not’.^41^ For the young suspect, then, the AA is someone older, who can answer questions the suspect may have or provide advice.^42^ In the 2013 case of *R (on the application of HC)*,^43^ where the courts considered the extension of the AA provision to 17-year-olds, the High Court stated that the AA was there to assist the young person ‘in the face of an intimidating criminal justice system’.^44^ Reiterating the Codes, the Court of Appeal viewed the AA as something that could minimise the risk that unreliable information would be provided by the accused ‘by seeing that the interview is properly and fairly conducted and by facilitating communication between the police and the suspect’.^45^ In practice, the AA may only be called for interview;^46^ however, the Court of Appeal deemed the AA’s presence as not simply limited to interview.^47^ In essence, the court, when deciding upon the admissibility of the evidence, is not simply considering whether the AA should have been present or whether the AA was suitable (as this is not sufficient for the exclusion of evidence); rather, they are assessing whether the AA’s absence or unsuitability renders the evidence inadmissible based on the PACE admissibility rules. Thus, their interpretation of admissibility relies on unreliability of confession evidence (section 76) or unfairness of admitting evidence (section 78) and not on whether the suspect should have had an AA or one that was suitable in the circumstances. If the evidence is excluded, the admissibility rules may offer the suspect some level of protection against, for example, wrongful conviction, yet the approach that the courts take is more akin to that of an evidential protection.

Whilst the limitations within the remedial framework were created by the legislature, the judiciary are not prevented from using their discretion (or, rather, interpretative judgment)^48^ when ruling upon admissibility. The courts could, for example, interpret as unreliable any confession obtained in breach of the Code C requirements under section 76 PACE or interpret as adversely impacting upon the fairness of the proceedings any evidence obtained in breach of the Code C requirements under section 78. Instead, the courts have often read these provisions rather restrictively; even where suspects have been unfairly or harshly treated by the police, the courts have still seen fit to admit the evidence. This can be seen in the case of *Nazish*,^49^ where the court dismissed as ‘fanciful’ the defendant’s claim of police oppression and the resultant impact on the reliability of his confession, despite the support of expert evidence.^50^ Similarly, in *Beeres*,^51^ the court failed to set aside the confession of the defendant, who was ‘quite drunk’ upon her arrest and, contrary to PACE, permitted less than eight hours’ sleep during detention. In cases relating to the non-implementation of the AA safeguard, the courts have taken a similar, although not always consistent, approach and have interpreted reliability narrowly.^52^

The courts have also used section 77—a jury direction which can be given where a ‘mentally handicapped’ suspect has confessed in the absence of an ‘independent person’ (taken to include a legal representative in addition to an AA)—to justify not excluding evidence under sections 76 and 78. This is despite section 77 being more restrictive in scope (requiring that the individual concerned is ‘mentally handicapped’ rather than ‘merely’ vulnerable) and requiring a lesser remedy than sections 76 and 78 (jury direction rather than exclusion of evidence). In the case of *Lewis*,^53^ for example, the court deemed section 78 to be of little relevance and, whilst accepting that the AA was absent, were not convinced that this called into question the reliability of the confession (under section 76). The court was not satisfied that the conviction rested wholly or substantially on the confession and further held that a solicitor could act as an independent person for the purposes of section 77 PACE.^54^

However, the courts have gone further than merely condoning breaches; they have also empathised with the police, such as in *Glaves*, where Owen J stated that it was ‘not always easy’ for police officers ‘to have every item of the Code in mind’ and that it was ‘of no consolation to the public at large that the police may be criticised’.^55^ Such could be viewed as reminiscent of the lack of criticism of the police during the Fisher Inquiry,^56^ and could lend further weight to the evidential safeguard contention. The AA’s function is not, however, simply limited by the Code or by the courts: it is also limited by the practical realities of who implements the safeguard and why, and who performs the role and for whom. In such instances, power dynamics may be at play.

### D. Practical Realities and Power Dynamics

As noted in the Introduction, the responsibility for implementing the safeguard is left with the custody officer or the investigative officer. Previous research has documented the issues with implementation, particularly for adult suspects: the police may exclude large swathes of the adult suspect population from the protection of the safeguard by adopting a narrow definition of vulnerability,^57^ may fail to identify whether a suspect is vulnerable^58^ and may decide not to call an AA because they do not think that the AA’s absence will be called into question at a later date.^59^ When deciding whether to call an AA, the officers principally consider whether the integrity of the evidence may be called into question. Yet, even where an AA is called, there may be problems with the safeguard itself, including: the quality of AAs^60^ or particular ‘types’ of AAs (such as volunteers, family members and friends, social workers or members of Youth Offending Teams (YOTs)); inconsistencies in service delivery;^61^ the functions of AAs;^62^ and the interpretation of the role and position of the AA.^63^ Further, the AA may be limited by power dynamics: similar to the solicitor, the AA, most notably for his or her safety, is dependent upon, and must therefore maintain a good working relationship with, the police.^64^ Thus, supposing that the AA knows when intervention may be required, the reality of having to navigate the power dynamics inherent within such relationships will invariably impact on the AA’s ability or willingness to intervene when the police are acting unfairly. Yet, the AA safeguard faces a more fundamental problem: it seeks to address multiple, and often conflicting, needs which exist across an array of different frameworks.

### E. Divergent Needs and Disparities

Whilst the AA safeguard appears only in the PACE Codes of Practice for adult suspects—and often only in the Notes for Guidance, which assume an even lower position ‘in terms of their authority’^65^ than the main body of the Code—provisions for young suspects can be found in legislation. The Crime and Disorder Act (CDA) 1998 requires YOTs^66^ to attend as AAs for young suspects where, for example, an AA known to the suspect, such as a family member, cannot otherwise be secured. This provision thus places the AA safeguard on a statutory footing for young suspects, creating disparities in how and whether the safeguard is implemented for young as compared with adult suspects, with the latter often at the mercy of the police officer’s decision making.^67^ Further, the safeguard, in seeking to address the vulnerabilities of both young and adult suspects, may further undermine the applicability of the safeguard for the latter. In previous research, custody officers were seen to apply the safeguard to children, but often limited it to adults who were ‘childlike’,^68^ with such categorisations often replicated in case law.^69^ This not only creates problems with implementation—where adults do not sufficiently ‘perform’ childlike characteristics, they may not be provided with an AA—but also runs the risk of infantilising adult suspects.

Further, there are differences within the adult suspect population: a suspect may be vulnerable because of a mental health condition, mental disorder, additional education need, neurodiverse condition, learning disability or difficulty, acquired brain injury or circumstance, or a combination thereof. The AA safeguard is not necessarily designed to address the needs of the suspect; indeed, it is intended to meet a vast array of different, and often divergent, needs. Taking the facilitation of communication as an example, given that the AA is not a specialist in autism spectrum condition (ASC) and, more importantly, how to meet the needs of someone with ASC (such as not being able to follow tagged or complex questioning), he or she may be limited when providing the facilitative link between the suspect and the police—or, indeed, anyone else. Even where the AA has been informed of specific needs, he or she may not have the expertise sufficient to address these needs. This issue may be ameliorated where the AA is known to the suspect and is aware of both the suspect’s needs and how to respond to them. However, in such cases, the AA will not necessarily have the legal and procedural knowledge required to protect the suspect. An AA from an organised scheme may be required to attend for adult suspects within these broad categories and to meet their undoubtedly complex and varied needs. Whilst specialism may be desired, it may be difficult to envisage a role where all eventualities and capabilities are covered. It is, however, both possible and preferable to aim to provide a safeguard that, at the very least, recognises the differences between children and adults. Before examining the potential of human rights and equalities frameworks to underpin the AA safeguard, it is worth acknowledging and exploring some alternative constructions of the AA safeguard and the limitations inherent within these constructions.

## 3. Social Constructions of the Safeguard

Pierpoint has examined how the AA’s role is interpreted by volunteer AAs and argued that the safeguard should be centred around welfare.^70^ Her work identified four threads to the AA’s role—crime control, due process, welfare and crime reduction. With the first two—crime control and due process—she relied upon Packer’s often discussed and debated value-types.^71^ For Pierpoint, crime control ‘prioritizes the conviction of the guilty, even at the risk of the conviction of some innocent people and with the cost of infringing civil liberties to achieve its goal’.^72^ For the AA’s role, crime control can be evidenced by the way in which the AA acts (for example, berating the suspect or being pro-police) or by the way in which the police approach the safeguard (for example, failing to advise the AA of his or her role). Due process ‘prioritizes the acquittal of the innocent, even at the risk of the frequent acquittal of the guilty. It is most concerned with protecting civil liberties and upholding values of reliability, equality and moral standards.’^73^ According to Pierpoint’s data, volunteer AAs espoused due process values such as ‘preventing unfair questioning, checking comprehension of questions and processes (such as the caution), and comforting the suspect’.^74^ Pierpoint further argues that the AA adhering to police procedures and reading the custody record is evidence of due process.^75^ She acknowledges, however, that much of this role lends itself more readily to ‘welfare’, which is taken to mean a response to the suspects’s emotional and physical needs.^76^ Volunteer AAs acted in a manner consistent with a welfare approach: they ensured that the suspect had food, water and adequate rest, and/or diverted the suspect ‘away from the harmful effects of custody’.^77^ Finally, Pierpoint addressed the crime prevention element of the AA’s role, whereby the AA would ‘[change] the social environments and motivations of potential or actual offenders to deter them from future offending’.^78^ Such an approach, as Pierpoint highlights, is explicitly recognised in section 37 of the CDA 1998, which requires that ‘all those involved in the youth justice system, [AAs included], prevent offending by young people’.^79^ In her study, volunteers would attempt to investigate and explore the suspect’s (alleged) offending.^80^ The problem with this, however, is that to address offending, an AA must presume guilt, thus taking him or her beyond the remit of the role and potentially placing the suspect in a difficult position if such presumptions lead the AA to encourage the suspect to confess.^81^

Whilst Pierpoint’s work is important when attempting to understand how volunteers interpreted their roles in respect of young suspects, she does not fully explore or critique the implications of each of the models she sets forth. Taking crime control, it is worth highlighting the disparities between how the safeguard operates for adults and children: whilst for both young and adult suspects the police are responsible for implementation, for young suspects the safeguard exists on a statutory basis, thus engendering greater compliance.^82^ Within crime control, efficiency, standardisation and routinisation are prioritised above suspects’ rights; the police are responsible for quality control; and safeguards are present to promote trust and confidence in the system without impeding the process.^83^ For the AA safeguard for adults, implementation is left to the police and is routine and minimal;^84^ remedial action must be sought through the courts (and few cases reach this stage);^85^ and the courts tend to be largely forgiving of police malpractice.^86^ This further highlights one of the two problems addressed in section 2E above—the imbalance for adults and children, and police responsibility for the implementation of the safeguard. Further, while the AA does have important welfare functions, this is arguably not the central purpose of the role; indeed, the welfare approach is complementary to the AA role, but is, in and of itself, insufficient when seeking to protect the rights and entitlements of the suspect or to (fully) safeguard the evidence.^87^ Pierpoint also does not go far enough in exploring the due process elements of the role: within this model, limits are placed on state power, the individual is placed at the forefront of the process, scrutiny and challenge are welcomed, and those who breach the rules are penalised by an acquittal of the accused, even where the evidence is otherwise reliable. Further, findings of legal guilt are only permitted where the rules have been followed and opportunities for appeal are open so long as there is any finding of impropriety, however small. As in Pierpoint’s research, the AA may facilitate due process;^88^ however, the AA safeguard is not framed in a manner consistent with a purely due process model. Further, Pierpoint’s research does not explore how the AA is conceptualised in law (as explored above) and how the AA’s role *could be* reframed (to which this article now turns). In section 4, it is proposed that the safeguard could be reframed through human rights and equalities frameworks.

## 4. Reframing the AA Safeguard: Resolving the Problems through Human Rights Frameworks

As argued above, the AA safeguard can be subject to multiple—and often conflicting—conceptualisations, but is principally constructed and operationalised as an evidential safeguard. Further, the safeguard tries to achieve too much, and in doing so fails to address the needs of the suspect and enable effective participation. Such problems could, at least in part, be resolved through human rights frameworks, and in this section the arguments for—and limitations of—such an approach will be set forth. Before doing so, it is worth acknowledging the developments in respect of vulnerability at an EU level under the ‘justice’ pillar. Within such developments, there is an imbalance between how adult suspects are protected as compared with young suspects: whilst a Directive^89^—which sets out the goals that EU countries should achieve (but does not dictate how countries must achieve these goals)—has required procedural safeguards for young suspects, a Recommendation^90^—which is non-binding—has been used to address the vulnerability of adults. Indeed, there has been no consensus at an EU level regarding the definition of vulnerability in respect of adults, and for this reason the roadmap will not be examined in further detail.^91^

### A. Article 6 ECHR

The requirement of effective participation is guaranteed through article 6 of the European Convention on Human Rights (ECHR), which recognises—through the broader right to a fair trial (a right that extends to pre-trial procedures^92^)—the principle of equality of arms. Article 6 requires that each party is ‘given a reasonable opportunity to present his case under conditions that do not place him at a disadvantage vis-à-vis his opponent’.^93^ The European Court of Human Rights (ECtHR) has previously recognised the ‘particularly vulnerable position’^94^ of the criminal suspect/defendant, also recognising a number of factors—chronic alcoholism, acute alcohol intoxication, a physical disability or medical condition, social disadvantage and/or mental disorder^95^—that could render a suspect vulnerable. Yet, the ECtHR did not propose how to address the vulnerability of the suspect, other than suggesting that a lawyer should be present. Whilst it should be acknowledged that all suspects may be ‘vulnerable’,^96^ there are unique circumstances in which this vulnerability may be enhanced and there should be additional protection offered to the suspect beyond that which is offered to all suspects.^97^ The right to a fair trial may thus inform the AA safeguard, but, given the lack of specific focus within article 6 on issues pertaining to vulnerability, the Convention alone is insufficient as an underpinning framework for the AA safeguard.

### B. The Equality Act 2010

Pursuant to the Equality Act 2010, the AA safeguard could be considered as a ‘reasonable adjustment’, which would enable the suspect to realise and enforce his or her fair trial rights.^98^ The Act requires that public bodies, including the police, consider the effect of their decisions and policies on those with protected characteristics.^99^ Relevant to the AA safeguard, section 20 of the 2010 Act requires the use of reasonable adjustments for people with disabilities.^100^ The nature of the adjustments relevant to the AA safeguard—the first and third requirements—can be found in section 20(3) and (4). The first requires the taking of reasonable steps to avoid disadvantage where a ‘provision, criterion or practice’ places a disabled person at a substantial disadvantage in comparison with a non-disabled person. The third requires that reasonable steps are taken to provide an auxiliary aid where the absence of the provision of that aid would place the disabled person at a disadvantage when compared with those who are not disabled. Yet, to avail of a section 20 adjustment, the individual must have a disability, defined as a ‘substantial and long-term adverse effect on [their] ability to carry out normal day-to-day activities’.^101^ This would therefore exclude claims for age-based adjustments as well as claims for adjustments based on circumstantial factors such as stress and bereavement, in addition to any condition not considered a ‘disability’. Such would exclude young suspects and non-disabled adults from the remit of the AA safeguard. The 2010 Act also leaves open the nature of the auxiliary aid and does not make clear who the decision maker should be. This would have to be determined and legislated for if the AA were to be considered a ‘reasonable adjustment’. If the section 20 duty were to rest upon the police, questions may arise regarding the AA’s independence. The Equality Act, whilst offering a basic framework for reform of the AA safeguard, is limited in both scope and application.

### C. International Human Rights: the UNCRPD and UNCRC

The safeguard for adult suspects could alternatively be conceptualised through the United Nations Convention on the Rights of Persons with Disabilities (CRPD). The CRPD is associated with a ‘new paradigm’ of disability rights, placing a strong emphasis on dignity, autonomy and independence, but rejecting concepts and values associated with charity, welfare and the medical model of disability. The CRPD was drafted with high levels of involvement of disabled people, who deliberately eschewed the language of ‘vulnerability’ in its development. To date, whilst the UNCRPD has prompted work on access to justice^102^ and civil law provisions to support the exercise of legal capacity (often known as ‘supported decision making’), and paid critical attention to criminal justice mechanisms such as unfitness to plead, the insanity defence and, most recently, guilty pleas,^103^ it has not been considered in relation to the AA safeguard within academic research.^104^

There are several CRPD articles that may be relevant to the AA safeguard. The first is contained in article 5, which relates to equality and non-discrimination, and places a duty on statutory agencies to make reasonable accommodation, as defined in article 2.^105^ The second right that is relevant for the AA safeguard is article 12, which requires equal recognition before the law; it places a duty on statutory agencies to provide support in the exercise of legal capacity, such support respecting the ‘rights, will and preferences’ of the person. Also relevant to the safeguard is article 13, relating to access to justice, which places a duty on statutory agencies to ensure ‘procedural and age-appropriate accommodations, in order to facilitate their effective role as direct and indirect participants, including as witnesses, in all legal proceedings, including at investigative and other preliminary stages’, and to promote ‘appropriate training for those working in the field of administration of justice’, including the police. Finally, article 14 of the CRPD requires that persons with a disability, if deprived of their liberty through any process, are treated ‘on an equal basis with others, entitled to guarantees in accordance with international human rights law’ and entitled to reasonable accommodation.

Whilst the CRPD holds some potential for reform of the AA safeguard, it is limited in its scope and application, applying only to disability and thus falling victim to the same problems as the Equality Act. As with the Equality Act, the CRPD may not extend to those who are considered ‘vulnerable’ for the purposes of the AA safeguard but are not considered to be disabled.^106^ Further, the CRPD approach, as with the Equality Act, leaves open the nature of the auxiliary aid and does not specify who should make the judgment when deciding whether to apply the aid. The statutory agency provision under article 12 also replicates another problem with the section 20 Equality Act duty: the police, if the statutory agency, would be responsible for provision of the AA safeguard, thus raising issues regarding independence. Moreover, the rights protected by the CRPD are, of course, not absolute, and would need to be balanced against competing rights for the person and others in society. However, the key benchmark for the CRPD is that the rights of disabled people are protected *on an equal basis with others*. As such, the critical issue here would be to identify comparators and thus consider any particular disadvantage that might be experienced by disabled people. A CRPD approach could thus inform the AA safeguard, albeit with recognition of the limitations outlined above. Crucially, the approach towards ‘disability’ would have to be broader in application to successfully underpin the AA safeguard.^107^

Thus far in this section, consideration has been given only to vulnerable adult suspects. Yet, it must also be acknowledged that young suspects also require the protection of an AA.^108^ As argued in section 2, it would be ideal—and, indeed, sensible—to consider separate frameworks for children and adults. At present, one safeguard seeks to meet the often divergent needs of adult and young suspects, but across two different ‘systems’—the adult criminal justice system and the youth justice system—with differing aims and objectives.^109^ The existence of a statutory safeguard for young suspects but not adults reflects the disjointed nature of the AA safeguard across these two systems.^110^ This also creates conflict and tension within, and obstacles to the facilitative nature of, the AA’s role.

A separate safeguard for young suspects could thus be conceptualised through the United Nations Convention on the Rights of the Child (CRC). The CRC urges that the best interests of the child—anyone below the age of 18^111^—be prioritised in any decisions and actions that affect children.^112^ Further, whilst the CRC, like the CRPD and Equality Act, does not comment on the adjustments to be made or the form they should take, it does require that any child who is accused of breaking the law is treated with dignity and respect, including having access to legal and any other appropriate assistance as well as to a fair trial, in a manner that accounts for their age. It can thus be argued that, in respect of young suspects, modifications are required to the criminal process, such as access to a specific safeguard. The CRC seeks ‘to protect and nurture childhood’ rather than ‘encourage equality for children with adults’,^113^ thus giving further weight to the contention that children—and, by extension, ‘young suspects’—and adults, and their needs, should be considered separately within procedural frameworks.

## 5. Conclusion

Within this article it has been argued that the AA safeguard is subject to multiple interpretations but that it principally operates as something through which the integrity of evidence is protected, often, but not always, to the detriment of the suspect. Further, it has been argued that the safeguard requires reform, which could potentially be achieved through human rights frameworks. In doing so, it is possible that some, but not all, of the problems with the safeguard could be remedied.

Much more is known today than in the 1980s, when this safeguard was introduced, not only about youth neuro- and emotional development, mental health, learning disabilities and difficulties, acquired brain injury and neurodiversity, but also about how these various aspects and characteristics can impact upon the suspect’s ability to participate in the criminal process.^114^ Whilst this article has examined the possibilities of human rights frameworks, much more work must be done in examining whether and, if so, how the safeguard could be reformed. For this, an explicit commitment to human rights and equality frameworks is required, and, crucially, questions must be raised regarding who is vulnerable and why, and exactly how the safeguard will address that. Following the CRC and CRPD, it may be preferable that due weight is given to the views of children and adults with disabilities in any discussion of reform.^115^

It is accepted that the AA safeguard is important in protecting suspects, ensuring—or seeking to ensure—effective participation and minimising miscarriages of justice, yet the doctrinal and empirical realities should serve as a cautionary tale to other jurisdictions seeking to introduce similar safeguards. Importantly, the first consideration should be: why is this safeguard being introduced and what does it aim to achieve? If the aim is effective participation, then human rights frameworks should be consulted, and possibly amended, to suit the specificities of the issue at hand. This article has taken the first step towards a reconceptualization of the safeguard, urging that the guidance must be reconsidered and rewritten, drawing upon—but not being beholden to—human rights frameworks. It would also be sensible to ensure that such guidance exists on a statutory footing to engender compliance and provide additional remedies for breach. The need for reconceptualisation and reform is ever more pressing as cases are, in effect, tried at the police station^116^ at a time when the criminal justice system is facing more pressure and less resource.^117^ It is imperative that effective and meaningful participation is secured at the earliest, and often only, stage of the criminal process.

## Footnotes

1 Home Office, *Code C Revised Code of Practice for the detention, treatment and questioning of persons by Police Officers* (TSO 2019); Home Office, *Code D Revised Code of Practice for the identification of persons by Police Officers* (TSO 2017); Home Office, *Code of Practice in connection with: The detention, treatment and questioning by Police Officers of persons in police detention under Section 41 of, and Schedule 8 to, the Terrorism Act 2000. The treatment and questioning by Police Officers of detained persons in respect of whom an authorisation to question after charge has been given under Section 22 of the Counter-Terrorism Act 2008* (TSO 2018); Home Office, *Code E Revised Code of Practice on audio recording interviews with suspects; Code F Revised Code of Practice on visual recording with sound of interviews with suspects* (TSO 2018).

2 The age of criminal responsibility in England and Wales and Northern Ireland is 10.

3 Defined as ‘any disorder or disability of the mind’ as per the Mental Health Act 1984 Code of Practice.

4 Roxanna Dehaghani and Chris Bath, ‘Vulnerability and the Appropriate Adult Safeguard: Examining the definitional and threshold changes within Code of Practice C to the Police and Criminal Evidence Act 1984’ [2019] Crim LR 213.

5 Home Office, *Code C 2019* (n 1) para 1.13(d).

6 ibid para 1.7.

7 ibid para 1.7.

8 ibid para 16.1; para 10.12; para 10.11A read alongside para 10.11; Annex A para 2B.11.17; para 1.4.

9 The definition of vulnerability has undergone significant revision—see Roxanna Dehaghani, *Vulnerability in Police Custody: Police Decision Making and the Appropriate Adult Safeguard* (Routledge 2019). Further, the AA, as will be explored later, is now performed by a vast array of individuals.

10 See John Jackson, ‘Responses to Salduz: Procedural Tradition, Change and the Need for Effective Defence’ (2016) 79 MLR 987.

11 TE St Johnston, ‘Judges’ Rules and Police Interrogation in England Today’ (1966) 57(1) J Crim L & Criminology 85, 85.

12 Christopher Price and Jonathan Caplan, *The Confait Confessions* (Marion Boyars 1977).

13 Henry Fisher, *Report of an Inquiry by the Hon. Sir Henry Fisher into the Circumstances Leading to the Trial of Three Persons on Charges Arising Out of the Death of Maxwell Confait and the Fire at 27 Doggett Road, London SE6* (HC 1977/78 90).

14 David Brown, Tom Ellis and Karen Larcombe, *Changing the Code: Police Detention Under the Revised PACE codes of Practice* (Home Office 1992) 70.

15 Royal Commission on Criminal Procedure, *The Royal Commission on Criminal Procedure Report* (Cmnd 8092, 1981). Otherwise known as the Phillips Commission.

16 As well as, for example, the introduction of the Prosecution of Offences 1985 and, thus, the establishment of the Crown Prosecution Service.

17 David Brown, ‘PACE Ten Years On: A Review of the Research’ (1997) Home Office Research Study No 155, 1–2.

18 The closest that PACE comes to providing a definition of vulnerability is the mention of the term ‘mentally handicapped’ in s 77. This provision—a jury direction (therefore only available in a Crown Court trial)—requires that the jury is advised that a ‘mentally handicapped’ person has confessed in the absence of an ‘independent person’. The independent person can be an AA, but could equally be an accredited or probationary representative, a probation worker or a healthcare professional.

19 Roxanna Dehaghani, ‘He’s Just Not That Vulnerable: Exploring the Implementation of the Appropriate Adult Safeguard in Police Custody’ (2016) 55 Howard Journal of Criminal Justice 396; Dehaghani, *Vulnerability in Police Custody* (n 9).

20 See Roxanna Dehaghani, ‘Defining the “Appropriate” in Appropriate Adult: Restrictions and Opportunities for Reform’ [2020] Crim LR 1133.

21 PACE, s 67(10).

22 Alternatively, in a trial on indictment (ie at the Crown Court), a direction may be given to the jury that the confession was given by a ‘mentally handicapped’ person in the absence of an ‘independent person’. However, this requires that (i) the person is deemed ‘mentally handicapped’ (a more restrictive definition than ‘vulnerable person’ under Code C) and (ii) no independent person was present (for the purposes of s 77, a solicitor or legal representative can also be an independent person).

23 PACE, s 76.

24 PACE, s 78.

25 Home Office, *Revised Code of Practice for the Detention, Treatment and Questioning of Persons by Police Officers. Police and Criminal Evidence Act (PACE) 1984. Code C* (Crown 2017) Note for Guidance 1G.

26 ibid Note 11C. See also Note for Guidance E2.

27 But see Dehaghani and Bath (n 4).

28 Mike McConville, Andrew Sanders and Roger Leng, *The Case for the Prosecution: Police Suspects and the Construction of Criminality* (Routledge 1991).

29 Home Office, *Code C 2019* (n 1) para 1.7.

30 This was arguably as a result of the input of the National Appropriate Adult Network (a charity that oversees AA provision across England, Wales, Northern Ireland and the Isle of Man). See Home Office and National Appropriate Adult Network, ‘Guide for Appropriate Adults’ (Home Office and NAAN 2011) <https://assets.publishing.service.gov.uk/government/uploads/system/uploads/attachment_data/file/117682/appropriate-adults-guide.pdf> accessed 3 September 2019; Home Office, ‘Guidance for Appropriate Adults’ (Home Office, 2003) <https://assets.publishing.service.gov.uk/government/uploads/system/uploads/attachment_data/file/117625/guidanceappadultscustody.pdf> accessed 5 November 2019.

31 Upon recommendation to the Home Office by the author of this article. The right to silence presents significant challenges: as Quirk has pointed out, lawyers are damned if they advise silence and damned if they do not—Hannah Quirk, *The Rise and Fall of the Right of Silence* (Routledge 2017). The AA, who may not be legally trained, may find this aspect of their role even more demanding and may therefore not, in practice, be able to ensure that the suspect’s right to remain silent is respected.

32
*A Local Authority v B* [2008] EWHC 1017 (Fam). See also Chris Bath, ‘Legal Privilege and Appropriate Adults’ (2014) 178(27) Criminal Law and Justice Weekly.

33 Dehaghani, *Vulnerability in Police Custody* (n 9).

34 Dehaghani, ‘Defining the “Appropriate”’ (n 20). See Home Office, *Code C 2018* (n 1) para 11.17A.

35 The author was alerted to this case through contact with the National Appropriate Adult Network. The author was asked to provide advice (from an academic perspective) on how the provisions could be interpreted. In this case, the AA was deemed by the police to be obstructive because he or she reminded the suspect of his or her right to legal advice during the police interview.

36 Harriet Pierpoint, ‘How Appropriate Are Volunteers as “Appropriate Adults” for Young Suspects?’ (2000) 22 JSWFL 383; Harriet Pierpoint, ‘A Survey on Volunteer Appropriate Adult Services’ (2004) 4(1) Youth Justice 32.

37 Jackie Hodgson, ‘Vulnerable Suspects and the Appropriate Adult’ [1997] Crim LR 785, 790 citing Dixon and others 1990; Harriet Pierpoint, ‘Extending and Professionalising the Role of the Appropriate Adult’ (2011) 33 JSWFL 139.

38 See Dehaghani, ‘Defining the “Appropriate”’ (n 20).

39
*R v Weekes* [1993] 97 Cr App R 222.

40 ibid 225.

41 ibid 227.

42 See Roxanna Dehaghani, ‘“Vulnerable by Law but Not by Nature”: Examining Child and Youth Vulnerability in the Context of Police Custody’ (2017) 39 JSWFL 454; Dehaghani, *Vulnerability in Police Custody* (n 9).

43
*R (on the application of HC) v The Secretary of State for the Home Department, The Commissioner of Police of the Metropolis* [2013] EWHC 982 (Admin).

44 ibid para 93.

45
*R v Campbell (Oliver Keith))* [1995] 1 Cr App R 522.

46 Dehaghani, *Vulnerability in Police Custody* (n 9).

47 See *R v Fogah* [1989] Crim LR 141; *R v Maguire* (1990) 90 Cr App R 115.

48 Simon Bronnit and Phillip Stenning, ‘Understanding Discretion in Policing’ (2011) 35 Crim LJ 319.

49
*R v Nazish (Muzaffer)* [2014] EWCA Crim 2947.

50 The defendant was an undiagnosed diabetic; he had not eaten and had consumed a mere two glasses of water during his 18-hour detention.

51
*Beeres v Crown Prosecution Service (West Midlands)* [2014] EWHC 283 (Admin).

52 Dehaghani, *Vulnerability in Police Custody* (n 9).

53 [1996] Crim LR 260.

54 The courts were correct to hold that a solicitor could act as an ‘independent person’ for the purposes of s 77 PACE but could be seen to have erred when interpreting the provisions under s 76 or 78.

55
*R v Glaves* [1993] Crim LR 685.

56 Fisher (n 13).

57 Dehaghani, *Vulnerability in Police Custody* (n 9).

58 ibid. See also eg Phillip Bean and Teresa Nemitz, *Out of Depth and Out of Sight* (University of Loughborough 1995); Gisli H Gudjonsson, Isobel Clare, Susan Rutter and John Pearce, ‘Persons at Risk During Interviews in Police Custody: The Identification of Vulnerabilities’ (1993) Royal Commission on Criminal Procedure Research Study No 12.; National Appropriate Adult Network, ‘There to Help: Ensuring Provision of Appropriate Adults for Mentally Vulnerable Adults Detained or Interviewed by Police’ (NAAN 2015); National Appropriate Adult Network, There to Help 2: Ensuring Provision of Appropriate Adults for Vulnerable Adults Detained or Interviewed by Police: An Update on Progress 2013/14 to 2017/18’ (NAAN 2019); Clare Palmer, ‘Still Vulnerable After All These Years’ [1996] Crim LR 633; Dehaghani, *Vulnerability in Police Custody* (n 9); Dehaghani, ‘He’s Just Not That Vulnerable’ (n 19); Dehaghani, ‘“Vulnerable by Law but Not by Nature”’ (n 42).

59 Dehaghani, *Vulnerability in Police Custody* (n 9).

60 Roger Evans, ‘Challenging a Police Caution Using Judicial Review’ [1996] Crim LR 104; Hodgson (n 37); Tricia Jessiman and Ailsa Cameron, ‘The Role of the Appropriate Adult in Supporting Vulnerable Adults in Custody: Comparing the Perspectives of Service Users and Service Providers’ (2017) 45 British Journal of Learning Disabilities 246; Vicky Kemp and Jackie Hodgson, ‘England and Wales: Empirical Findings’ in Miet Vanderhallen and others (eds), *Interrogating Young Suspects: Procedural Safeguards from an Empirical Perspective* (Intersentia 2016); Brian Littlechild, ‘Reassessing the Role of the “Appropriate Adult”’ [1995] Crim LR 540; Pierpoint, ‘How Appropriate’ (n 36); Katie Quinn and John Jackson, ‘Of Rights and Roles: Police Interviews with Young Suspects in Northern Ireland’ (2007) 47 Brit J Criminol 234; Ciaran White, ‘Re-assessing the Social Worker’s Role as an Appropriate Adult’ (2002) 24 JSWFL 55.

61 Mark Perks, ‘Appropriate Adult Provision in England and Wales: Report Prepared for the Department of Health and the Home Office’ (NAAN 2010) <www.gov.uk/government/uploads/system/uploads/attachment_data/file/117683/appropriate-adult-report.pdf> accessed 28 February 2019.

62 Hodgson (n 37); Quinn and Jackson (n 60); Pierpoint, ‘How Appropriate’ (n 36); White (n 60).

63 Her Majesty’s Inspectorate of Constabulary (HMIC), *The Welfare of Vulnerable People in Police Custody* (HMIC 2015); Pierpoint, ‘A Survey’ (n 36); Phillip Fennell, ‘Mentally Disordered Suspects in the Criminal Justice System’ (1994) 21 J Law & Soc 57; Brown (n 17); Dehaghani, *Vulnerability in Police Custody* (n 9).

64 See Quirk (n 31).

65 Michael Zander, *The Police and Criminal Evidence Act 1984* (6th edn, Sweet & Maxwell 2013).

66 These are multi-agency teams which are coordinated by a local authority to reduce reoffending amongst children and young people.

67 Dehaghani, *Vulnerability in Police Custody* (n 9).

68 ibid.

69 ibid.

70 Harriet Pierpoint, ‘Reconstructing the Role of the Appropriate Adult in England and Wales’ (2006) 6 Criminology and Criminal Justice 219. Pierpoint’s research has examined various aspects of the AA’s role, such as: professionalisation (Pierpoint, ‘Extending and Professionalising’ (n 37)); delays to securing AAs (Harriet Pierpoint, ‘Quickening the PACE: The Use of Volunteers as Appropriate Adults’ (2008) 18 *Policing and Society* 397); variations across Youth Offending Teams (YOTs) in terms of service delivery and thus the nature and quality of the AA’s role (Pierpoint, ‘A Survey’ (n 36)); and volunteer performance and the overall intentions of the AA safeguard (Harriet Pierpoint, ‘The Performance of Volunteer Appropriate Adults: A Survey of Call Outs’ (2001) 40 Howard Journal of Criminal Justice 255), particularly their selection and preparation, where she also touched upon human rights implications under the European Convention on Human Rights and the Human Rights Act 1998 (Pierpoint, ‘How Appropriate’ (n 36). It is worth noting, however, that Pierpoint’s work focuses on young suspects only and thus does not consider the AA, including its interpretation, in relation to adults.

71 Herbert L Packer, *The Limits of the Criminal Sanction* (Stanford UP and Oxford UP 1968). For discussion, see David J Smith, ‘Case Construction and the Goals of the Criminal Process’ (1997) 37 Brit J Criminol 335; Mike McConville, Andrew Sanders and Roger Leng, ‘Descriptive or Critical Sociology: The Choice is Yours’ (1997) 37 Brit J Criminol 347; Peter Duff, ‘Crime Control, Due Process and “The Case for the Prosecution”’ (1998) 38 Brit J Criminol 611.

72 Pierpoint, ‘Reconstructing’ (n 70) 221.

73 ibid.

74 ibid 225.

75 ibid.

76 ibid 221.

77 ibid 222.

78 ibid.

79 ibid.

80 ibid.

81 ibid. Where the suspect has offended, the crime prevention function of the safeguard may prove useful in providing the suspect with assistance in addressing such offending, provided this is not done in a ‘responsibilising’ manner—see Dehaghani, ‘“Vulnerable by Law but Not by Nature”’ (n 42) for a discussion of responsibilisation in the context of the AA safeguard and young suspects. For a discussion on responsibilisation more generally, see David Garland, *The Culture of Control: Crime and Social Order in Contemporary Society* (University of Chicago Press 2001).

82 Dehaghani, *Vulnerability in Police Custody* (n 9).

83 Pierpoint thus includes managerialism within her discussion.

84 Dehaghani, *Vulnerability in Police Custody* (n 9).

85 Jackson (n 10).

86 But see Pierpoint, ‘Reconstructing’ (n 70) 221.

87 The welfare aspect of the role may also be limited because AAs are not always present for the entire custody process.

88 Pierpoint, ‘Reconstructing’ (n 70).

89 Directive (EU) 2016/800 of European Parliament and the Council on procedural safeguards for children who are suspects or accused persons in criminal proceedings [2016] OJ L132/1.

90 European Commission proposal for a Directive of the European Parliament and of the Council on procedural safeguards for children suspected or accused in criminal proceedings, COM (2013) 822 final, 3.

91 Also worth noting is the decision taken by the UK opt-out of the EU roadmap on the basis that it purportedly transferred too much protection to suspects at the expense of victims—see Lore Mergaerts and Roxanna Dehaghani, ‘Protecting Vulnerable Suspects in Police Investigations in Europe: Lessons Learned from England & Wales and Belgium’ (2020) 11 NJECL 313.

92
*Teixeira de Castro v Portugal* (1998) EHRR 101.

93 European Court of Human Rights, Guide on Article 6 of the European Convention on Human Rights: Right to a Fair Trial (Criminal Limb) <www.echr.coe.int/Documents/Guide_Art_6_criminal_ENG.pdf> accessed 3 September 2019, citing *Öcalan v Turkey* [GC], § 140; *Foucher v France*, § 34; *Bulut v Austria*; *Faig Mammadov v Azerbaijan*, § 19.

94
*Salduz v Turkey* App no 36391/02 (ECtHR 27 November 2008). See also *Panovits v Cyprus* App no 4268/04 (ECtHR, 11 December 2008); *Shabelnik v Ukraine* App no 16404/03 (ECtHR, 19 February 2009); *Pishchalnikov v Russia* App no 7025/04 (ECtHR, 24 September 2009); *Dayanan v Turkey* App no 7377/03 (13 October 2010).

95
*Plonka v Poland* App no 20310/02 (ECtHR, 31 March 2009); *Bortnik v Ukraine* App no 39582/04 (ECtHR, 27 January 2011); *Orsus and others v Croatia* App no 15766/03 (EctHR, 16 March 2010); *Borotyuk v Ukraine* App no 33579/04 (EctHR, 16 December 2010).

96 Roxanna Dehaghani, ‘Interrogating Vulnerability: Reframing the Vulnerable Suspect in Police Custody’ (2021) 30 *Social and Legal Studies* 251.

97 See ibid, where it is argued (through Fineman’s vulnerability theory—see eg Martha Albertson Fineman, ‘The Vulnerable Subject and the Responsive State’ (2010) 60 Emory LJ 1) that all suspects are indeed vulnerable and that instead the question should be whether suspects have resilience.

98 See also NAAN, ‘There to Help’ (n 58).

99 See ch 1 of the Equality Act 2010 for a full list of protected characteristics.

100 There are three requirements under this duty, two of which are relevant to the AA safeguard. The other—physical features that place a disabled person at a disadvantage compared with non-disabled persons—will not be considered here.

101 Equality Act 2010, s 6(1).

102 Eilionóir Flynn, *Disabled Justice? Access to Justice and the UN Convention on the Rights of Persons with Disabilities* (Routledge 2015). See also Anna Lawson and Eilionóir Flynn, ‘Disability and Access to Justice in the European Union: Implications of the United Nations Convention on the Rights of Persons with Disabilities’ in Lisa Waddington, Gerard Quinn and Eilionóir Flynn (eds), *Yearbook of European Disability Law 3* (Intersentia 2013) 7–44.

103 See Jill Peay and Elaine Player, ‘Pleading Guilty: Why Vulnerability Matters’ (2018) 81 MLR 929.

104 The potential for the CRPD was acknowledged for the first time (to the author’s knowledge) in NAAN, ‘There to Help’ (n 58) paper B.

105 There are clear parallels with s 20 of the Equality Act 2010.

106 See Dehaghani, *Vulnerability in Police Custody* (n 9).

107 It is worth noting that the most recent version of Code C utilises a social model of disability in respect of vulnerability and the AA safeguard, rather than the much-critiqued medical model—see Dehaghani and Bath (n 4).

108 Young suspects may have a disability that further compounds their inability to effectively participate in the pre-trial criminal process; further consideration must be given to how to address the multiple vulnerabilities of some suspects.

109 Children are typically—although not always, as in the well-reported case of Thompson and Venables—processed through the youth justice system, which is, at least purportedly, less punitive than the adult system. As Muncie has noted, the (youth) criminal justice response may favour ‘punitive values’ rather than ‘protection and support’: John Muncie, ‘The “Punitive Turn” in Juvenile Justice: Cultures of Control and Rights Compliance in Western Europe and the USA’ (2008) 8 Youth Justice 107, 110. The youth justice system has also tended to responsibilise children: see John Muncie, ‘Governing Young People: Coherence and Contradiction in Contemporary Youth Justice’ (2006) 26 Critical Social Policy 770, 711. Bateman has noted that there has been a shift away from punitiveness between 2008 and 2015 ‘driven by financial imperatives’: Tim Bateman, ‘The State of Youth Justice 2015: An Overview of Trends and Developments’ (NAYJ 2015) 2 http://thenayj.org.uk/wp-content/uploads/2015/10/State-of-Youth-Justice-Oct15.pdf.

110 Indeed, the extension of the AA safeguard to cover 17-year-olds in *HC* sought to bring the safeguard in line with the rest of the youth justice system.

111 CRC, art 1.

112 CRC, art 3.

113 James Boyden, ‘Childhood and Policy Makers: A Comparative Perspective on the Globalization of Childhood’ in Allison James and Alan Prout (eds), *Constructing and Reconstructing Childhood* (Falmer Press 1997) 199.

114 See Dehaghani, *Vulnerability in Police Custody* (n 9).

115 In respect of children and children with disabilities, see CRC, art 12; CRPD, art 7(3); more generally, see the CRPD, which views persons with disabilities as subjects who are capable of making their own decisions rather than objects of, *inter alia*, social protection.

116 Jackson (n 10).

117 See eg Roxanna Dehaghani and Daniel Newman, ‘The Crisis in Legally Aided Criminal Defence in Wales: Bringing Wales into Discussions of England and Wales’ (2021) 41 LS 234; Daniel Newman and Roxanna Dehaghani, *Experiences of Criminal Justice: Perspectives from Wales on a System in Crisis* (Bristol University Press forthcoming).

